# Comparative tissue transcriptomics reveal prompt inter-organ communication in response to local bacterial kidney infection

**DOI:** 10.1186/1471-2164-12-123

**Published:** 2011-02-21

**Authors:** Jorrit Boekel, Örjan Källskog, Monica Rydén-Aulin, Mikael Rhen, Agneta Richter-Dahlfors

**Affiliations:** 1 Swedish Medical Nanoscience Center, Department of Neuroscience, Karolinska Institutet, Retzius väg 8, SE-171 77 Stockholm, Sweden; 2 Department for Medical Cell Biology, Biomedical Center, Uppsala University, Box 571, SE-751 23 Uppsala, Sweden; 3 Department of Microbiology, Tumor and Cell Biology, Karolinska Institutet, Nobels väg 16, SE-171 77 Stockholm, Sweden

## Abstract

**Background:**

Mucosal infections elicit inflammatory responses via regulated signaling pathways. Infection outcome depends strongly on early events occurring immediately when bacteria start interacting with cells in the mucosal membrane. Hitherto reported transcription profiles on host-pathogen interactions are strongly biased towards *in vitro *studies. To detail the local *in vivo *genetic response to infection, we here profiled host gene expression in a recent experimental model that assures high spatial and temporal control of uropathogenic *Escherichia coli *(UPEC) infection within the kidney of a live rat.

**Results:**

Transcriptional profiling of tissue biopsies from UPEC-infected kidney tissue revealed 59 differentially expressed genes 8 h post-infection. Their relevance for the infection process was supported by a Gene Ontology (GO) analysis. Early differential expression at 3 h and 5 h post-infection was of low statistical significance, which correlated to the low degree of infection. Comparative transcriptomics analysis of the 8 h data set and online available studies of early local infection and inflammation defined a core of 80 genes constituting a "General tissue response to early local bacterial infections". Among these, 25% were annotated as interferon-γ (IFN-γ) regulated. Subsequent experimental analyses confirmed a systemic increase of IFN-γ in rats with an ongoing local kidney infection, correlating to splenic, rather than renal *Ifng *induction and suggested this inter-organ communication to be mediated by interleukin (IL)-23. The use of comparative transcriptomics allowed expansion of the statistical data handling, whereby relevant data could also be extracted from the 5 h data set. Out of the 31 differentially expressed core genes, some represented specific 5 h responses, illustrating the value of comparative transcriptomics when studying the dynamic nature of gene regulation in response to infections.

**Conclusion:**

Our hypothesis-free approach identified components of infection-associated multi-cellular tissue responses and demonstrated how a comparative analysis allows retrieval of relevant information from lower-quality data sets. The data further define marked representation of IFN-γ responsive genes and a prompt inter-organ communication as a hallmark of an early local tissue response to infection.

## Background

Host responses to bacterial infections are dynamic, well-controlled processes of high complexity, and are utterly important in protecting the body. Molecular understanding of signaling mechanisms orchestrating the innate immune response is required to define new targets for future treatments of bacterial infections. In mucosal infections, bacterial recognition and the earliest immune signaling occur at the epithelium, which can alert the surrounding tissue by producing cytokines [1-3]. Resident immune cells are activated, as is the endothelium of nearby vessels. As a consequence, additional immune cells, recruited from the blood stream, extravasate to the site of infection to directly or indirectly aid in the eradication of bacteria. Clearance of infection is commonly accompanied by dramatic alterations of tissue architecture and function, and involves processes such as coagulation, hypoxia, ischemia, edema, and scarring [4-6].

Recent development of tools enabling intravital imaging of the host's organs is shedding new light on the molecular understanding of processes underlying tissue responses [7-9]. Real-time studies within the organ of a live animal have contributed to our understanding of bacterial infection in kidney tubules [6,10], spirochete behavior in the vasculature [11], immune cell migration in granulomas [12], and transcription factor activation in bovine mastitis [13]. Alongside the visualization, quantitative real-time (q-RT) PCR can be performed on dissected tissues, enabling quantification of *in vivo *expression of selected host genes [6], and specific bacterial virulence factors, such as the toxin α-hemolysin [10].

Transcription profiling studies have conveyed considerable advantages in delineating complex host responses [14]. The power of transcriptomics lies in its hypothesis-free nature, which makes it an ideal tool to discover unknown processes. Also, the sheer number of measurements contained in each transcriptomic experiment enables data clustering to yield stronger results. Cell culture experiments have for long been the primary source for transcriptomic information [15]. The well-defined nature of such experiments allows detailed molecular studies of specific events. On the contrary, transcriptomes of *in vivo *infection models have been little researched, possibly due to current technical limitations and because the complexity of the model systems negatively impacts analytical ease. A sparse number of infection models with live bacteria are reported, using *Shigella flexneri *[16], *Listeria monocytogenes *[17] and *Escherichia coli *[18,19] as infectious agents. Also, transcriptomic analyses have been performed using LPS as stimulus [20-22].

We have previously established a protocol for intravital, real-time imaging of uropathogenic *E. coli *(UPEC) -induced pyelonephritis in the exposed kidney of a live rat [10,23]. Slow infusion of bacteria directly into the lumen of the proximal tubule allows the infection process to be studied with high spatial and temporal resolution, in the presence of all of the live animal's physiological factors, such as the vascular, nervous, immune and hormonal systems. We found bacterial colonization to be a very rapid process, which brought about major changes in tissue homeostasis. Tissue oxygen tension dropped to 0 mm Hg within 3-4 h, followed by clotting and cessation of vascular flow in peri-tubular capillaries. Localized ischemia was identified as an innate immune defense mechanism as it protected bacteria from gaining entry into the systemic circulation, thus protecting the host from sepsis. While bacteria were contained at the infection site, immune cell recruitment occurred. Their active participation in host cell signaling processes resulted in bacterial clearance and localized tissue edema within 24 h.

In this study, we analyze the nature of the network-like signal transduction pathways that orchestrate the pronounced tissue reactions. A whole-genome based transcriptomic and bioinformatic approach, using tissue dissected at 3, 5, and 8 h post-infection, was applied to study the molecular details governing the intra- and inter-organ communication. Comparative transcriptomics defined a general core of genes upregulated as an early response to infection, which guided us towards the identification of unexpected systemic responses.

## Results

### Dynamics of a single nephron-infection

**Figure 1:**
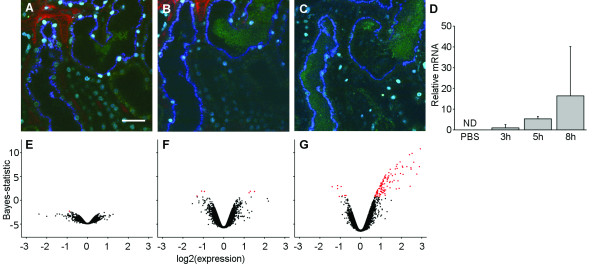
**Bacterial growth and tissue response within the first 8 h post-infection**. Proximal tubule of rat kidney infected by UPEC. A-C. Imaging by two-photon microscopy of infected kidney 3 h (A), 5 h (B), and 8 h (C) post-infection. Bacteria are shown in green, blood flow is labelled with red dextran, injected tubules are visualized by endocytosed blue dextran. Scale bar 30 μm. D. Relative expression of *gfp+ *by bacteria quantified by qRT-PCR. Measurements are normalized to the 3 h time point. ND - not detected. Error bars show SD, n = 3. E-G. Gene expression 3 h (E), 5 h (F), and 8 h (G) post-infection quantified by microarray. Each point represents one array probe, n = 3. Probes marked in red have adjusted *P-*values of <0.59 (E), <0.15 (F), and <0.05 (G).

The real-time dynamic events accompanying an early kidney infection (3-8 h) within a living host were studied using the GFP^+^-expressing derivative, LT004, of UPEC strain CFT073 [10,24]. The same infection protocol was applied as previously described [10]. In short, bacteria were slowly infused into the lumen of a superficial proximal tubule in the kidney of an anaesthetized rat. Two-photon microscopy was then applied to image the progression of infection within the organ. Starting from a few bacteria adherent to the epithelial lining, tubular colonization could be followed during the first 8 h of the infection process. Figure 1A-C shows a time-dependent increase in luminal green fluorescence, indicative of bacterial multiplication. As the infecting bacterial strain expresses *gfp*+ from a constitutive promoter, a positive correlation between *gfp+ *transcript abundance and bacterial numbers was assumed. To investigate this, the injection site was excised from rats sacrificed 3 h, 5 h, or 8 h after the injection of either UPEC or PBS. Following RNA extraction, *gfp+ *transcript was quantified using qRT-PCR. Although levels of the transcript varied between rats, an increase in *gfp+ *expression during the course of infection was apparent (Figure 1D). As expected, no *gfp+ *transcript was detected in PBS-injected control rats.

During this early time course of infection, the growing number of bacteria was well contained within the lumen of the proximal tubule (Figure 1A-C). Yet, the colonization process is associated with rapid alterations of tissue homeostasis in the surrounding renal parenchyma, i.e. loss of peritubular capillary flow, and ischemia [6,10]. Further molecular knowledge is required to understand how signals are relayed from the initial bacterium/epithelium point-of-contact, to other cells in the organ and onwards, to other tissues in the host.

### Overview of transcriptome analyses

To study the cross-talk that orchestrates the hosts' tissue response to infection, we took an approach to define the reprogramming the host transcriptome undergoes at the very site of infection. To achieve this, RNA was purified from the infection site at 3 h, 5 h, and 8 h post-infection and used for transcriptional profiling by a two-color microarray covering 22,012 rat genes. The source for RNA extractions was standardized, such that 40-60 mm^3 ^renal cortex was dissected from each animal, containing either the infected or the PBS sham-operated nephron. Preprocessed data from microarrays was first subjected to multiple testing [25], and differential expression was determined by the use of adjusted *P-*value and fold-expression thresholds. To evaluate the validity of the data, the annotated functions of the identified genes were analyzed according to their Gene Ontology (GO) biological process characteristics. Possible overrepresentation of any particular protein function was analyzed using the BINGO software [26,27]. The GO analysis allowed us to lower the threshold, yet obtaining valid data from the analysis. We used the same principle to perform comparative transcriptomic analyses, in which generated data was aligned to other early (<12 h) host-response data sets available online. This enabled us to lower the differential expression thresholds while still retaining stringency of the analysis. The procedure thus allowed us to reliably detect genes that were commonly expressed in multiple data sets. All different analyses, and the data retrieved, are summarized in Table 1.

**Table 1:** Amount of probes or genes differentially expressed at different thresholds

Data set	p^a ^< 0.05		p < 0.05 & |fold exp|>2		p < 0.5		B^b^>-2.5	
	probes	genes	probes	genes	probes	genes	probes	genes

3 h post-infection	0	0	0	0	-	-	-	-
5 h post-infection	0	0	0	0	-	-	368	-
8 h post-infection	113	-^c^	76	59	458	341	386	-
Study *b*^d^	-	-	81	61	11208	-	1480	-
Study *c*	-	-	86	71	569	-	722	-
Study *d*	-	-	363	265	11961	-	3660	-

^a ^Adjusted *P*-value

^b ^Bayes statistic

^c ^not determined

^d ^For reference see Table 2

### The transcriptome in response to early renal infection and functional assessment of differentially expressed genes

**Figure 2:**
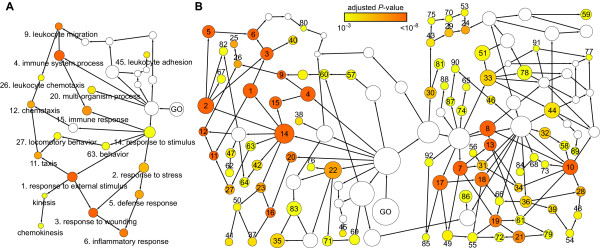
**Clustering of differentially expressed host genes into functionally defined categories**. GO annotations of differentially expressed genes identified at 8 h post-infection were analyzed and visualized in a directed acyclic graph using the BINGO plugin in Cytoscape. Categories are shown as circles, the diameter corresponding to the amount of found genes in a category. The adjusted *P*-value for overrepresentation of differentially expressed genes was calculated for each category and color coded, white circles have no overrepresentation. Circles marked GO represent the root node of gene ontology categories and contain all found genes. A. Genes retrieved with the criteria adjusted *P*-value <0.05 and differential expression ≥2. The numbering corresponds to the numbers in B. B. Genes retrieved with the criterion adjusted *P*-value <0.5. Numbering represents GO categories, see supporting information S4. The left half of the graph includes all numbered categories in Figure 2A, the right half comprises categories involved in biological regulation.

For probes whose IDs were present in the ENSEMBL database, adjusted *P*-values were calculated. We found that the lowest adjusted *P*-value for a differentially expressed probe in the 3 h sample was 0.58 (Figure 1E), whereas 6 probes were identified 5 h post-infection with adjusted *P*-values ranging between 0.12 and 0.15 (Figure 1F). The 8 h sample yielded 113 unique probes that showed differential expression with adjusted *P*-values < 0.05 (Figure 1G, Table 1). Adding the criterion that the expression ratio should be ≥ 2 yielded 76 unique probes whose ENSEMBL IDs could be mapped to 59 *bona fide *Entrez genes (Table 1, Additional file 1 Table S1). To decipher which tissue responses were associated with early, local bacterial infections, we analyzed the annotated functions of the 59 identified genes according to their GO characteristics. This showed that most identified gene products clustered in several categories, all relating to "immune response" (Figure 2A, Additional file 2 Table S2).

Inherent to the infection model used in this study is that biopsies from which RNA was prepared contain 1000 - 2000 uninfected nephrons in addition to the infected one. It can thus be assumed that the resulting signal-to-noise ratio may introduce a risk of overlooking genes whose induction falls below the thresholds "adjusted *P*-values < 0.05" and "expression ratio ≥ 2". To increase the amount of relevant information from the 8 h microarray data set, the GO analysis was repeated, but this time using an adjusted *P*-value < 0.5. As expected, this procedure expanded the list of differentially expressed probes from 113 to 458. The 458 probes mapped to 341 Entrez genes (Table 1, Additional file 3 Table S3). While this list is likely to contain numerous false positive entries due to low threshold, we argued that these entries would be randomly dispersed in the cluster analysis, rather than enriched in specific GO categories. Hence, we compared the color code of the cluster circles representing the extended list of 341 genes (Figure 2B, Additional file 4 Table S4) to those originating from the 59 genes in the previous analysis (Figure 2A, Additional file 2 Table S2). The observed shift from yellow to red in a subset of cluster circles indicated a statistical strengthening of those GO categories. Moreover, an expansion of categories was observed, including those related to the "immune response", as well as the appearance of a new group, representing processes related to "biological regulation".

A closer inspection of results presented in Figure 2 identified many categories with gene products expected to participate in the defense against bacterial infections. This included clusters of proteins involved in inflammatory responses, such as response to bacteria, chemotaxis, and leukocyte migration, thus confirming several of the tissue responses previously implicated in early, local bacterial infections [10]. Moreover, Figure 2 revealed that numerous genes coding for functions associated with cell death, apoptosis, proliferation, response to hypoxia, and wound healing had become activated, reflecting the apparent versatility of the host response to a local infection.

### A common core of genes expressed during early local inflammation

**Figure 3:**
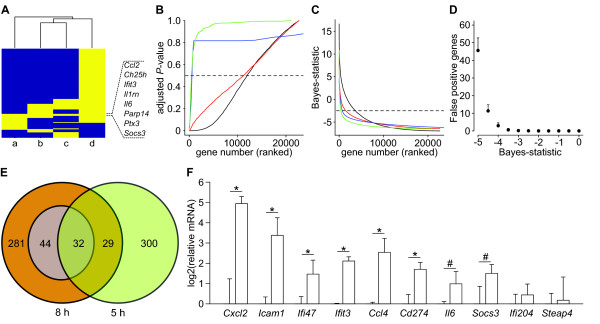
**Commonly upregulated genes during early local inflammation**. A. Hierarchical clustering of differentially expressed genes (rows) from studies a-d (columns) (Table 2). Yellow represents differentially expressed genes, while blue are non-differentially expressed. Genes differentially expressed in all datasets are shown in text. B and C. Genes in each study were ranked after adjusted *P*-value (B) or Bayes statistic (C), the rank number was plotted against adjusted *P-*value (B) and Bayes statistic (C). Study a - green, b - blue, c - red, and d - black. The dashed horizontal line shows an adjusted *P-*value of 0.5 (B) or a Bayes statistic of -2.5 (C). D. Gene names in all four studies were replaced with random names. Amounts of differentially expressed genes present in all four studies were plotted against Bayes statistic threshold. Error bars show SD of 20 randomized trials. E. Venn diagram showing the relationship between probes differentially expressed (Bayes statistc ≥-2.5) at 8 h (orange) and 5 h (green), respectively. The purple circle represents probes differentially expressed 8 h post-infection with adjusted *P*-value <0.05 and fold expression >2. Amounts of probes are given. F. Relative expression levels of core genes quantified by qRT-PCR. Expression levels in UPEC- (white bars) and PBS -injected tissue were normalized to that of PBS injected-tissue. *indicate a *P*-value <0.05 defined by Student's t-test between PBS and UPEC injected samples, # indicate a *P*-value <0.10. Error bars show SD, n = 3.

To evaluate the biological relevance of the transcriptomic data, we compared our results to those reported for other models that used Gram-negative stimuli. To match the experimental conditions as close as possible to our infection model, the following criteria were applied: the transcriptome had to be analyzed at *early time points *(within 12 h) after *local delivery *of the stimuli into an otherwise *sterile organ*. A database search identified three studies that fulfilled these criteria, all based on administration of LPS (Table 2, studies *b-d*). To allow the comparison of data originating from our rat model to those performed in mice, the rat gene nomenclature was converted to the corresponding homologous mouse genes. The comparative analysis of the four data sets, illustrated by the heat-map presented in Figure 3A, indicated that 8 genes are upregulated in all four studies (adjusted *P*-value < 0.05, expression level ≥ 2). Hence, the genes *Ccl2, Ch25 h, Ifit3, Il1rn, Il6, Parp14, Ptx3*, and *Socs3 *can be defined as a common core of genes expressed early in response to local infection and inflammation caused by Gram-negative stimuli.

**Table 2:** Microarray data sets analyzed in this study

Study	Animal	Organ	Agent	Delivery	Challenge time (h)	Reference
*a*	Rat	Kidney	*E. coli *CFT073	Tubular injection	8	E-MEXP-2136 This work
*b*	Mouse	Brain	LPS	Intraparenchymal	12	E-GEOD-6509 [20]
*c*	Mouse	Brain	LPS	Intracerebroventricular	6	E-MEXP-420 [21]
*d*	Mouse	Lung	LPS	Aerosol inhalation	4	E-GEOD-2411 [22]

The GO analysis demonstrated that its scientific validity was retained when one threshold was lowered, provided other statistical criteria were maintained. This encouraged us to lower the adjusted *P*-value threshold for gene expression, while retaining the criterion for genes to be present in all data sets, to see if the common core could be expanded. When the adjusted *P*-value threshold was altered from 0.05 to 0.5 and 'expression level ≥ 2' criterion was removed, we found a moderate increase of genes (~500 probes) in studies *a *and *c*, whereas a dramatic increase of approximately 12,000 probes was observed in studies *b *and *d *(Figure 3B, Table 1). This uneven distribution of probes posed a risk of introducing false positive entries into the core. To circumvent this, an analysis was performed using the Bayes statistics of genes [28], since we found that this method generated a more even distribution of probes among the 4 studies (Figure 3C, Table 1). To decide the appropriate Bayes statistic threshold to be used, a random comparative analysis was modeled. Figure 3D shows that as few as 0.05 false positive probes would be detected when applying a Bayes statistic threshold ≥ -2.5, while lower cutoffs considerably increased the risk of false positive entries. When applying a Bayes statistic threshold ≥ -2.5 in the comparative analysis, 386 differentially expressed probes were generated from data set *a *(Figure 3C, Table 1, Additional file 3 Table S3), whereas the corresponding numbers for data sets *b*, *c*, and *d*, were 722, 1480, and 3660 probes, respectively (Figure 3C, Table 1). When comparing the lists, 80 genes were found to be present in all 4 data sets, and almost all of these were relevant for infection and inflammation (Table 3). It thus appears that lowering of the threshold is valid provided other criteria are applied.

**Table 3:** Core of commonly expressed genes 8 h post-infection

Gene	Function
*Serpine1*	serine (or cysteine) peptidase inhibitor, clade E, member 1
*Nfkbia*	nuclear factor of kappa light polypeptide gene enhancer in B-cells inhibitor, alpha
*Oasl1*	2'-5' oligoadenylate synthetase-like 1
*Ifi44*	interferon-induced protein 44
*Cxcl2*	chemokine (C-X-C motif) ligand 2
*Tifa*	TRAF-interacting protein with forkhead-associated domain
*Ccl3*	chemokine (C-C motif) ligand 3
*Selp*	selectin, platelet
Zfp36 *	zinc finger protein 36
*Irf7*	interferon regulatory factor 7
Ifi205 *	interferon activated gene 205
*Birc3*	baculoviral IAP repeat-containing 3
*Myd88*	myeloid differentiation primary response gene 88
*Ccl19*	chemokine (C-C motif) ligand 19
Socs3 *	suppressor of cytokine signaling 3
*Nfkbie*	nuclear factor of kappa light polypeptide gene enhancer in B-cells inhibitor, epsilon
*Ier3*	immediate early response 3
*Pfkfb3*	6-phosphofructo-2-kinase/fructose-2,6-biphosphatase 3
*Ccl4*	chemokine (C-C motif) ligand 4
*Ptx3*	pentraxin related gene
*Il1b*	interleukin 1 beta
*Glipr2*	GLI pathogenesis-related 2
*Arid5a*	AT rich interactive domain 5A (Mrf1 like)
Cxcl10 *	chemokine (C-X-C motif) ligand 10
Ifit3 *	interferon-induced protein with tetratricopeptide repeats 3
*Cxcl1*	chemokine (C-X-C motif) ligand 1
Irg1 *	immunoresponsive gene 1
*Gadd45b*	growth arrest and DNA-damage-inducible 45 beta
*Parp14*	poly (ADP-ribose) polymerase family, member 14
*Serpina3n*	serine (or cysteine) peptidase inhibitor, clade A, member 3N
Ifi47 *	interferon gamma inducible protein 47
*Il1rn*	interleukin 1 receptor antagonist
*Rgs16*	regulator of G-protein signaling 16
*Il6*	interleukin 6
*Akap12*	A kinase (PRKA) anchor protein (gravin) 12
*Upp1*	uridine phosphorylase 1
*Icam1*	intercellular adhesion molecule 1
*Slc15a3*	solute carrier family 15, member 3
*Tmem2*	transmembrane protein 2
*Oas1 g*	2'-5' oligoadenylate synthetase 1G
Sod2 *	superoxide dismutase 2, mitochondrial
*Ifih1*	interferon induced with helicase C domain 1
*BC006779*	cDNA sequence BC006779
*Steap4*	STEAP family member 4
*Parp9*	poly (ADP-ribose) polymerase family, member 9
*Slfn4*	schlafen 4
*Timp1*	tissue inhibitor of metalloproteinase 1
*Plek*	pleckstrin
*Nfkb2*	nuclear factor of kappa light polypeptide gene enhancer in B-cells 2, p49/p100
*Ptges*	prostaglandin E synthase
*Ripk2*	receptor (TNFRSF)-interacting serine-threonine kinase 2
Cflar *	CASP8 and FADD-like apoptosis regulator
*Cebpb*	CCAAT/enhancer binding protein (C/EBP), beta
Cxcl11 *	chemokine (C-X-C motif) ligand 11
*Rcan1*	regulator of calcineurin 1
*Hk2*	hexokinase 2
*Ccl2*	chemokine (C-C motif) ligand 2
*Pvr*	poliovirus receptor
Cd274 *	CD274 antigen
*Tubb6*	tubulin, beta 6
*Ch25h*	cholesterol 25-hydroxylase
Irf1 *	interferon regulatory factor 1
*Slc7a2*	solute carrier family 7 (cationic amino acid transporter, y+ system), member 2
*Trib1*	tribbles homolog 1 (Drosophila)
*Vcam1*	vascular cell adhesion molecule 1
*Pde4b*	phosphodiesterase 4B, cAMP specific
*S100a9*	S100 calcium binding protein A9 (calgranulin B)
Psme2 *	proteasome (prosome, macropain) 28 subunit, beta
Psmb9 *	proteasome (prosome, macropain) subunit, beta type 9 (large multifunctional peptidase 2)
Lcn2 *	lipocalin 2
*Lcp1*	lymphocyte cytosolic protein 1
*Cdkn1a*	cyclin-dependent kinase inhibitor 1A (P21)
Cp *	ceruloplasmin
Ptgs2 *	prostaglandin-endoperoxide synthase 2
Psmb8 *	proteasome (prosome, macropain) subunit, beta type 8 (large multifunctional peptidase 7)
Hck *	hemopoietic cell kinase
*Lif*	leukemia inhibitory factor
*Nfkb1*	nuclear factor of kappa light polypeptide gene enhancer in B-cells 1, p105
Cxcl9 *	chemokine (C-X-C motif) ligand 9
Stat1 *	signal transducer and activator of transcription 1

*Genes induced by IFN-γ

### A comparative core analysis extracts relevant information from the 5 h data set

Lowering the threshold in comparative studies provides a possibility to retrieve information from data sets originally believed to be of too low significance. This prompted us to apply this method to the transcriptome data from the 5 h post-infection sample, first by comparing it to the 8 h data set. When a Bayes statistic threshold ≥ -2.5 was applied, 361 differentially expressed probes were identified in the 5 h data set (Table 1, Additional file 5 Table S5), as compared to 386 probes found in the 8 h data set (Table 1, Additional file 3 Table S3). By comparing the two data sets (Figure 3E), 61 probes were identified as differentially expressed at both time points. The majority of these are likely to be true positive entries, as the probability of finding the same probes in two random data sets of these sizes is limited to 5. Our analysis thus demonstrates that approximately 56 probes are differentially expressed at both time points.

To analyze whether a common core host response can be identified as early as 5 h, the 5 h data set was compared to the 3 studies listed in Table 2 (studies *b*-*d*). By applying a similar strategy as previously described, this analysis generated a core of 31 differentially expressed genes (Table 4). Studying their annotated protein function revealed that these core genes primarily are involved in processes linked to the inflammatory response. In addition, a subset of genes was found at 5 h but not at 8 h. This illustrates that the dynamic nature of gene regulation in response to bacterial infections can be addressed using comparative transcriptomics.

**Table 4:** Core of commonly expressed genes 5 h post-infection

Gene	Function
*Oasl1*	2'-5' oligoadenylate synthetase-like 1
*Cxcl2*	chemokine (C-X-C motif) ligand 2
*Ccl3*	chemokine (C-C motif) ligand 3
*Mx1*	myxovirus (influenza virus) resistance 1
*Irf7*	interferon regulatory factor 7
*Maff*	v-maf musculoaponeurotic fibrosarcoma oncogene family, protein F (avian)
*Birc3*	baculoviral IAP repeat-containing 3
*Cmpk2*	cytidine monophosphate (UMP-CMP) kinase 2, mitochondrial
*Cxcl10*	chemokine (C-X-C motif) ligand 10
*Ifit3*	interferon-induced protein with tetratricopeptide repeats 3
*Cxcl1*	chemokine (C-X-C motif) ligand 1
*Parp14*	poly (ADP-ribose) polymerase family, member 14
*Serpina3n*	serine (or cysteine) peptidase inhibitor, clade A, member 3N
*Il1rn*	interleukin 1 receptor antagonist
*Icam1*	intercellular adhesion molecule 1
*Slc15a3*	solute carrier family 15, member 3
*Oas1g*	2'-5' oligoadenylate synthetase 1G
*Tnfrsf12a*	tumor necrosis factor receptor superfamily, member 12a
*Parp9*	poly (ADP-ribose) polymerase family, member 9
*Slfn4*	schlafen 4
*Timp1*	tissue inhibitor of metalloproteinase 1
*Nfkb2*	nuclear factor of kappa light polypeptide gene enhancer in B-cells 2, p49/p100
*Ripk2*	receptor (TNFRSF)-interacting serine-threonine kinase 2
*Cxcl11*	chemokine (C-X-C motif) ligand 11
*Pml*	promyelocytic leukemia
*Irf1*	interferon regulatory factor 1
*Trib1*	tribbles homolog 1 (Drosophila)
*S100a9*	S100 calcium binding protein A9 (calgranulin B)
*Ccl7*	chemokine (C-C motif) ligand 7
*Ptgs2*	prostaglandin-endoperoxide synthase 2
*Lif*	leukemia inhibitory factor

### Experimental verification of core gene expression

Since core genes were partly derived from statistically insecure data, they needed to be experimentally verified. To test the reliability of differentially expressed genes defined as members of the common core host response at 8 h post-infection (Table 3), 10 genes were randomly selected and their induction tested using qRT-PCR. Primers specific for their corresponding rat sequences (Additional file 6 Table S6) were used to analyze the same mRNA preparations that had acted as sources for the microarrays. Figure 3F shows that *Cxcl2*, *Icam1, Ifi47, Ifit3, Ccl4*, and *Cd274 *could be verified as upregulated at a significance level of *P *<0.05 in the infected tissue, whereas expression of *Il6 *and *Socs3 *was upregulated at significance levels of *P *= 0.059 and *P *= 0.075, respectively. A statistically verified upregulation of *Ifi204 *and *Steap4 *could not be confirmed by qRT-PCR, although a trend towards upregulation was observed.

### IFN-γ responses during early host responses

It is interesting to notice that as many as 21 of the 80 core genes in the 8 h data set are annotated as IFN-γ inducible (Table 3). Compared to an estimated presence of 2% IFN-γ inducible genes in the human genome [29,30], the large proportion (>25%) in our data set demonstrates a significant overrepresentation (Fisher's exact test, *P *= 1.24 × 10^-15^).

**Figure 4:**
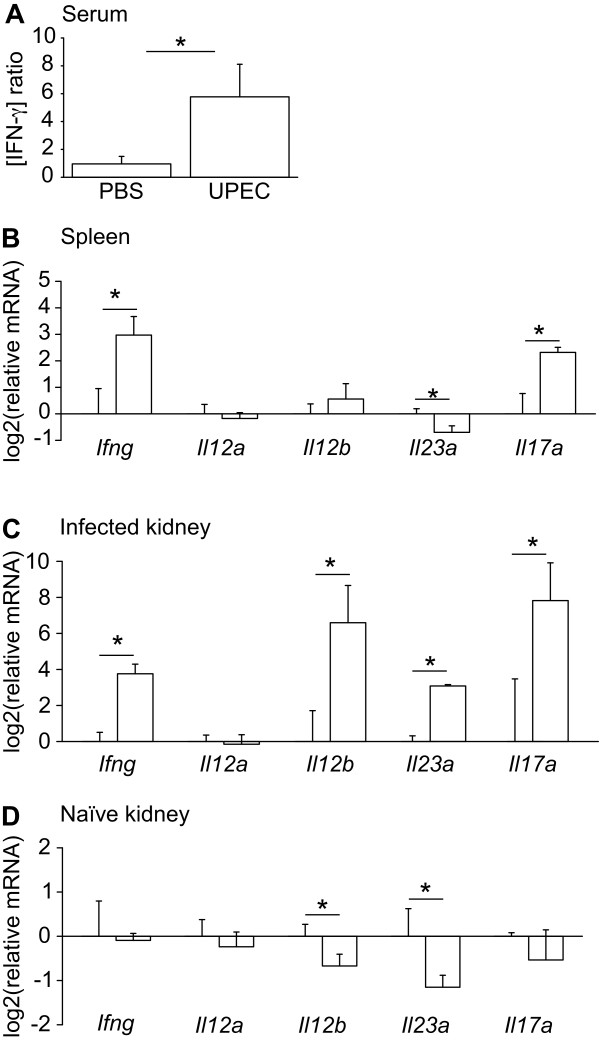
**IFN-γ responses to early infection. UPEC or PBS were injected into rat kidney proximal tubules**. A. Ratio of IFN-γ concentration at 8 h relative to 0 h, measured by ELISA, in serum from PBS or UPEC injected animals. * *P*-value <0.05 defined by a Student's t-test between PBS and UPEC injected animals log-transformed ratios of IFN-γ concentrations at 8 h to 0 h. B-D. Relative expression of different genes in spleen and kidney measured by qRT-PCR 8 h post-infection. Expression levels in UPEC (white bars) and PBS samples were normalized to that of PBS samples. * *P*-value <0.05 defined by Student's t-test between samples from PBS- and UPEC-injected animals. Gene expression in spleen tissue (B), kidney tissue that has been injected with UPEC or PBS (C), and naïve kidney tissue located ~1 cm from the injected site (D). In all panels, error bars show SD, n = 3.

One site in the body that acts as a source of IFN-γ production is the spleen. If this major secondary lymphoid organ produces IFN-γ during early kidney infections, increased serum levels can be assumed. This was tested by ELISA assays, which revealed a 5- to 6-fold increase of IFN-γ in the blood from animals 8 h post-infection as compared to blood samples taken just prior to infection. In contrast, no change in IFN-γ serum levels was observed in PBS sham-injected animals during the same time period (Figure 4A). When the relative amount of *Ifng *transcript in the spleen from animals with an ongoing renal UPEC infection was measured 8 h post-infection, *Ifng *transcript was indeed upregulated (Figure 4B). This was in contrast to spleen from PBS sham-injected animals. The role of cytokines IL-12 and IL-23 for induction of IFN-γ has previously been described [31,32]. Since IL-12 and IL-23 are heterodimers of IL-12a/IL-12b and IL-12b/IL-23a, respectively, we quantified the relative expression of the corresponding genes in spleen tissue in an attempt to identify the signals responsible for splenic IFN-γ production. qRT-PCR revealed that none of *Il12a*, *Il12b*, or *Il23a *were upregulated (Figure 4B), suggesting that neither IL-12 nor IL-23 are produced in the spleen. Yet, it appeared that the spleen was affected by inflammatory signaling, since IL-23's downstream target *Il17a *[33] was upregulated in the spleen of infected animals.

The expression levels of the four cytokines were also quantified at the renal infection site. Both IL-23 subunits *Il12b *and *Il23a *as well as down-stream target genes *Ifng *and *Il17a *were found to be upregulated in UPEC-infected kidney tissue, while no apparent upregulation of *Il12a *was observed (Figure 4C). To investigate whether this expression pattern represents a general renal response, naïve kidney tissue, located ~1 cm distant from either the UPEC or the PBS injection sites, was also analyzed. We observed that *Il12b *and *IL23a *were downregulated while no effect on other tested genes was seen (Figure 4D). Collectively, these data suggest that IL-23 is formed at the infection site, which in turn may induce local renal as well as splenic expression of IFN-γ and IL-17.

## Discussion

Clearance of bacterial infections poses great challenges for the vertebrate host. While there is an obvious need to rapidly eradicate bacteria from the infection site, unnecessary collateral tissue damage associated with inflammation must be avoided. This requires a balanced response, which is likely to change its character during different phases of the infection process. Results from the transcriptomics analysis corroborated this reasoning. The significance of transcriptional responses at different time points, measured as adjusted *P-*values, correlated to the progression of infection. Low significance was obtained 3 h post-infection when few bacteria were present in the proximal tubule and no apparent tissue damage was observed. In contrast, statistically significant data were generated 8 h post-infection when bacteria had efficiently colonized the tubule and immune cells had infiltrated the site. Our previous findings, based on intravital imaging and physiological recordings, demonstrate however that pronounced local tissue activities already occur within the first 3-4 h of infection [6,10]. This discrepancy may reflect the difficulty to apply a transcriptomics approach to complex tissues, and explains why it has preferentially been used in *in vitro *cell culture systems.

The infection protocol used in this paper was based on administration of washed overnight UPEC cultures. Clearly, such a culture includes a fraction of dead bacteria and LPS in addition to the live UPEC. Initially, LPS and dead bacteria may interact/bind to the epithelium, thereby inducing an inflammatory response. However, with the rapid growth of live bacteria these effects must be considered transient in the perspective of our analysis.

Appropriate biopsies for the study of dynamic tissue responses demand a strict control of the infection in space and time. In the current rat model, only one out of ca. 30,000 nephrons is infected. The infected nephron must be analyzed together with some immediately surrounding tissue to allow inclusion of peritubular capillaries and cells extravasated from the blood stream. Dissected biopsies contained ~1,500 uninfected nephrons in addition to the infected one. Yet, the microarray analysis identified 59 genes to be differentially expressed 8 h post-infection. In light of the vast number of uninfected nephrons, sheer detection of differential expression implies that cells in and around the bacteria-exposed nephron elicit a strong response to infection. Most of the differentially expressed genes were found to be upregulated. This may be attributed to the steady state gene expression in the uninfected nephrons, which may efficiently mask any downregulation. Also, mRNA from recruited immune cells contributes specifically to the pool of upregulated genes. Thus, the few downregulated genes identified in this study may result from a general downregulation in cells throughout the renal cortex.

Functional classification revealed an overrepresentation of differentially expressed genes in GO categories related to the "immune response". Although not a surprising result, it illustrates the validity of our approach. A previous finding, showing that ischemia is induced as a host response during renal colonization [6], was corroborated by the present analysis, as genes coding for proteins involved in wound healing and hypoxia were identified. This, and the fact that numerous genes involved in "biological regulation" were identified, underscores the complexity of the tissue response to infection.

Comparative transcriptomics can be used to retrieve information from microarray data sets that are of too low significance when treated individually. The power of comparative transcriptomics was shown by Jenner and Young when they, based on 32 studies involving 77 different types of host-pathogen interactions, defined 511 genes as a 'Generic alarm signal' to infection [15]. The present analysis applied more stringent parameters when selecting data sets. This may explain why the common core of "General tissue response to early local bacterial infections" was limited to 80 genes. While 39 of these genes also were present in the core of 'Generic alarm signal', 41 were not. The latter group may represent a response specifically obtained in the live animal model of infection, in the presence of all influencing physiological factors.

Using comparative transcriptomics, significant data could also be retrieved from the 5 h data set, both by comparing to the 8 h data set, and by building a core out of the 5 h data set and data sets *b-d*. Approximately 60 genes were differentially expressed at both 5 h and 8 h post-infection. The 5 h post-infection core of "General tissue response to early local bacterial infections" included 31 genes. This indicates that a significant induction occurs already at 5 h post-infection that is maintained over the following hours. In contrast, a sub-set of genes in the 5 h core were not induced at 8 h, which suggests a short-lived, time-dependent activation. Collectively, this demonstrates the feasibility of the present approach to study the dynamics of tissue responses during infection.

Closer inspection of the common core of "General tissue response to early local bacterial infections" revealed that ca 25% of the genes was associated to IFN-γ responses. IFN-γ is a key mediator of inflammation and immunity, controlling the balance between bacterial clearance on the one hand, and limitation of tissue damage as a consequence of inflammation on the other [34]. As the common core predicted, we could detect significant amounts of IFN-γ in the systemic circulation of rats with an ongoing local kidney infection. Several roles for systemic IFN-γ have been reported. Immune cells, such as neutrophils and monocytes, become primed already in the bloodstream, and may therefore act more efficiently once they reach the site of infection [35]. For example, IFN-γ activates neutrophil defense systems, such as phagocytosis and production of reactive oxygen species [35,36], as well as induction of monocyte- and T cell-attracting chemokine production [37]. Conversely, systemic IFN-γ is also known to downregulate IL-8 and matrix metalloproteinase production, thereby downregulating recruitment of neutrophils [34,35]. Thus, systemic IFN-γ may have dual roles in the UPEC infection, balancing the appropriate inflammatory response while restricting tissue damage.

At present, we can only speculate about the inter-organ communication leading to splenic IFN-γ production. The infection kinetics of the local infection may limit the recruitment of IFN-γ producing cells into the renal tissue. It is thus unlikely that the infected kidney acts as the source for serum IFN-γ. Yet, the attracted immune cells will participate in the production of other cytokines, which may act as triggers for IFN-γ production. We excluded the classical IFN-γ inducing cytokine IL-12, since *Il12a *transcripts were not upregulated in either kidney or spleen. Instead, our results suggest a possible signaling cascade where a local renal IL-23 production induces IFN-γ in the spleen. IL-23 dependent IFN-γ production has previously been demonstrated by others [32]. Alternatively, IL-23 may act indirectly via IL-17, which we found upregulated in both renal and spleen tissue. This hypothesis is supported by the finding that IL-23 induces IL-17 expression in γδ T cells in the spleen [33], as well as a recent report, showing IL-17 dependent IFN-γ production in a renal ischemia-reperfusion model [38]. IL-17 has previously been shown to be involved in the host defense to urinary tract infections [39]. Further research is however necessary to resolve a possible causality between these cytokines.

The host thus mobilizes the entire circulation even for a small infection, a process analogous to the acute phase response that affects the entire animal upon infection [40]. We speculate that in renal infections, inter-organ communication leading to elevated systemic signals may be advantageous in preventing future infections at other sites. Numerous examples exist demonstrating the ability of the kidney to cross-talk with other organs [41]. Acute kidney injury, *i.e. *ischemia, can induce inflammatory cascades in other organs leading to organ failure and mortality, sometimes within hours ([41] and references therein). The series of molecular events we report are also remarkably fast: between initiation of bacterial colonization and systemic presence of IFN-γ lie less than 8 h. Collectively, data presented herein highlight that gaining a full understanding of the infection process requires innate immune responses to preferentially be studied in live animal models.

## Conclusions

In this report we have investigated the transcriptome of a local kidney infection at three early time points. The differential expression signal increased over time, and an array of different host responses could be identified 8 h post-infection. These responses not only included up-regulated immune response genes but in addition genes for regulatory pathways defining the complexity of the response in intact tissue. A comparative transcriptomics approach identified 80 genes common for different *in vivo *models of acute infections and inflammations, suggesting a core response independent of infected tissue. Some of these common genes have no apparent function coupled to infection/inflammation, making them candidates for future research. Yet, a quarter of the activated genes pointed to a systemic IFN-γ response where the cytokine would be produced in the spleen where *Ifng *transcription was found strongly induced. The host thus mobilizes the entire circulation even for a local infection, a process analogous to the acute phase response that affects the entire animal upon infection. It thereby follows, that the series of molecular events we report are remarkably fast; the amount of time between bacterial colonization, systemic presence of IFN-γ as well as subsequent IFN-γ-mediated gene inductions lies within 8 h. The elevated IFN-γ serum levels observed may imply important dual roles for this cytokine in balancing the inflammatory response to clear the infection, while at the same time restricting tissue damage. Thus, we speculate that inter-organ communication leading to elevated systemic signals may be advantageous in preventing future infections at other sites. Results presented here thus show that the integrated system that live animal models offer is essential for the study of early host responses.

## Methods

### Bacteria and animals

The clinical isolate UPEC strain LT004 (CFT073, cobS::Φ(P_LtetO-1_-*gfp+*), Cm^R^; O6:K2:H1) [10] was grown in Luria-Bertani (LB) [42] medium supplemented with chloramphenicol (20 μg/ml) at 37°C. Male Sprague-Dawley (259 ± 20 g) (Scanbur BK, Sweden) rats with free access to chow and water were used. Studies have been approved by Uppsala djurförsöksetiska nämnd (Sweden) and the Institutional Animal Care and Use Committee (Indianapolis, IN, USA).

### Microperfusion and two-photon microscopy

Microperfusion was done as described by Melican *et al. *[6] with some modifications. Rats were anaesthetized by intraperitoneal injection of thiobutabarbital (Inactin^®^; 120 mg/kg) (Sigma-Aldrich, Sweden). Animals had a tracheostomy and cannulation of the femoral artery for measurement of arterial blood pressure and blood sampling, the femoral vein for infusion of Ringer's solution (5 ml/kg/h), and left ureter to divert renal output and bacteria away from the bladder. Dyes were injected via a jugular vein cannulation. Core body temperature was monitored rectally and maintained using heating pads. Strain LT004 was grown overnight, washed with PBS, and concentrated to 10^9 ^cfu/ml in PBS with 1 mM CaCl_2_, 2 mM MgCl_2_, and 1 mg/ml Fast Green FCF (Eastman Kodak, Rochester, NY, USA). For two-photon experiments, 0.2 mg/ml cascade blue-conjugated 10 kDa dextran (Molecular Probes, Eugene, OR, USA) was added. Bacterial suspensions or PBS were infused at a rate of 40 nl/min over 10 min into the lumen of superficial proximal tubules using a micromanipulator and a microinfusion pump. Two-photon microscopy was performed as in Melican *et al. *[6].

### Microarray

Tissue was isolated from whole kidney using a 5 mm biopsy punch with medullar tissue removed from the sample. Total RNA extraction was done on ~30 mg tissue using Trizol reagent (Invitrogen, Sweden), and purified with RNEasy Mini kit (QIAGEN, Sweden), including an on-column DNA digestion step. cDNA was synthesized from 10 μg RNA, using random primers and Superscript III RT enzyme (Invitrogen, Sweden). The cDNA was labeled with either Cy3 or Cy5 dyes (GE Healthcare, Sweden) by amino-allyl dye coupling, and hybridized to KTH Microarray Center's KTH Rat 27 k Oligo Microarray (ArrayExpress accession number A-MEXP-554 at http://www.ebi.ac.uk/microarray-as/ae/). Hybridization was done in Pronto Long oligo/Universal Hybridization solution (Corning, Corning, NY, USA) at 42°C for 20 h. Slides were scanned using a Genepix 4000B scanner (Axon Instruments, MDS Analytical Technologies, Sunnyvale, CA, USA) and data processed with Genepix Pro software (Axon Instruments). All data analysis was done in the R environment for statistical computing, using Bioconductor software [43] and the kth package [44] in R. For data preprocessing, raw two-color array data were filtered on saturation, size, and median-to-mean ratios. Bad spots were removed, and data were print-tip lowess normalized. Array results have been submitted to the ArrayExpress database under accession number E-MEXP-2136.

### Cluster analysis

Entrez genes identified in the microarray were analyzed using the Cytoscape [26] plugin BINGO [27] which relies on GO annotation of genes [45]. Overrepresentation of GO terms was tested using a hypergeometric test that was corrected for multiple testing, using a Benjamini and Hochberg correction [25].

### Processing of data from other microarray analyses

To extract differentially expressed genes, downloaded data sets were subjected to a limma analysis [28] similar to that applied to our own data. Extracted genes were clustered using a binary distance measure.

### Quantitative real time PCR

RNA was extracted from a small volume of dissected spleen or from kidney as described above. cDNA was transcribed from 1 μg RNA using SuperScript III First Strand Synthesis Supermix kit (Invitrogen). PCR was done using a 7500 Real Time PCR System (Applied Biosystems, Sweden) and Power SYBR Green PCR Mastermix (Applied Biosystems). In all experiments, *Gapdh *was used as reference gene. Primer sequences are listed in additional file 6 Table S6.

### ELISA assay

Blood was sampled at indicated time points from rats, and serum was isolated by centrifugation after coagulation, samples were diluted 1:20. The assay was done using 96-well Nunc Maxisorp plates (Nunc, Denmark) and a sandwich ELISA kit for rat IFN-γ (Mabtech, Sweden) according to the manufacturer's instructions.

## Abbreviations

GO: Gene Ontology; IFN: interferon; IL: interleukin; qRT-PCR: quantitative real time PCR; UPEC: uropathogenic *E. coli*

## Authors' contributions

JB performed experiments, participated in animal experiments, performed microarray data analysis, participated in study design, and participated in the writing of the paper. ÖK performed animal experiments. MRA participated in the writing of the paper. MR and ARD guided the study design and wrote the paper. All authors read and approved the final manuscript.

## Supplementary Material

Additional file 1 Table of differentially expressed genes (thresholds adjusted *P*-value 0.05, fold expression >2) in rat kidney 8 h post-infection Click here for file

Additional file 2 **Output of BINGO analysis of Entrez gene ID's of bona fide annotated genes found in additional file **1 Click here for file

Additional file 3 Table of differentially expressed genes (threshold adjusted *P*-value 0.5) in rat kidney 8 h post-infection Click here for file

Additional file 4 **Output of BINGO analysis of Entrez gene ID's found in additional file **3 Click here for file

Additional file 5 Table of differentially expressed genes (threshold Bayes > -2.5) in rat kidney 5 h post-infection Click here for file

Additional file 6 Table of primer sequences used in qRT-PCR Click here for file

## References

[B1] Bäckhed F Söderhäll M Ekman P Normark S Richter-Dahlfors A Induction of innate immune responses by *Escherichia coli *and purified lipopolysaccharide correlate with organ- and cell-specific expression of Toll-like receptors within the human urinary tract Cell Microbiol 2001 3 153 158 11260138 10.1046/j.1462-5822.2001.00101.x

[B2] Medzhitov R Preston-Hurlburt P Janeway CA Jr A human homologue of the Drosophila Toll protein signals activation of adaptive immunity Nature 1997 388 394 397 10.1038/41131 9237759

[B3] Hagberg L Hull R Hull S McGhee JR Michalek SM Svanborg Eden C Difference in susceptibility to gram-negative urinary tract infection between C3H/HeJ and C3H/HeN mice Infect Immun 1984 46 839 844 6389367 10.1128/iai.46.3.839-844.1984 PMC261623

[B4] Papadopoulos MC Verkman AS Aquaporin-4 gene disruption in mice reduces brain swelling and mortality in pneumococcal meningitis J Biol Chem 2005 280 13906 13912 10.1074/jbc.M413627200 15695511

[B5] Mak RH Kuo H-J Pathogenesis of urinary tract infection: an update Curr Op Pediatrics 2006 18 148 152 10.1097/01.mop.0000193276.39495.0d 16601494

[B6] Melican K Boekel J Månsson L Sandoval R Tanner G Källskog Ö Palm F Molitoris B Richter-Dahlfors A Bacterial infection-mediated mucosal signalling induces local renal ischaemia as a defence against sepsis Cell Microbiol 2008 10 1987 1998 10.1111/j.1462-5822.2008.01182.x 18549455

[B7] Ashworth SL Sandoval RM Tanner GA Molitoris BA Two-photon microscopy: visualization of kidney dynamics Kidney Int 2007 72 416 421 10.1038/sj.ki.5002315 17538570

[B8] Denk W Delaney KR Gelperin A Kleinfeld D Strowbridge BW Tank DW Yuste R Anatomical and functional imaging of neurons using 2-photon laser scanning microscopy J Neurosci Methods 1994 54 151 162 10.1016/0165-0270(94)90189-9 7869748

[B9] Eichhoff G Busche MA Garaschuk O *In vivo *calcium imaging of the aging and diseased brain Eur J Nucl Med Mol Imaging 2008 35 Suppl 1 S99 106 10.1007/s00259-007-0709-6 18193219

[B10] Månsson L Melican K Boekel J Sandoval R Hautefort I Tanner G Molitoris B Richter-Dahlfors A Real-time studies of the progression of bacterial infections and immediate tissue responses in live animals Cell Microbiol 2007 9 413 424 16953802 10.1111/j.1462-5822.2006.00799.x

[B11] Moriarty TJ Norman MU Colarusso P Bankhead T Kubes P Chaconas G Real-time high resolution 3D imaging of the lyme disease spirochete adhering to and escaping from the vasculature of a living host PLoS Pathogens 2008 4 e1000090 10.1371/journal.ppat.1000090 18566656 PMC2408724

[B12] Egen JG Rothfuchs AG Feng CG Winter N Sher A Germain RN Macrophage and T cell dynamics during the development and disintegration of mycobacterial granulomas Immunity 2008 28 271 284 10.1016/j.immuni.2007.12.010 18261937 PMC2390753

[B13] Notebaert S Carlsen H Janssen D Vandenabeele P Blomhoff R Meyer E *In vivo *imaging of NF-kB activity during *Escherichia coli*-induced mammary gland infection Cell Microbiol 2008 10 1249 1258 10.1111/j.1462-5822.2008.01123.x 18241210

[B14] Waddell SJ Butcher PD Stoker NG RNA profiling in host-pathogen interactions Curr Op Microbiol 2007 10 297 302 10.1016/j.mib.2007.05.013 PMC3128493 17574903

[B15] Jenner RG Young RA Insights into host responses against pathogens from transcriptional profiling Nat Rev Microbiol 2005 3 281 294 10.1038/nrmicro1126 15806094

[B16] Fernandez MI Regnault B Mulet C Tanguy M Jay P Sansonetti PJ Pédron T Maturation of paneth cells induces the refractory state of newborn mice to *Shigella *infection J Immunol 2008 180 4924 4930 18354217 10.4049/jimmunol.180.7.4924

[B17] Lecuit M Sonnenburg JL Cossart P Gordon JI Functional genomic studies of the intestinal response to a foodborne enteropathogen in a humanized gnotobiotic mouse model J Biol Chem 2007 282 15065 15072 10.1074/jbc.M610926200 17389602

[B18] Reigstad CS Hultgren SJ Gordon JI Functional genomic studies of uropathogenic *Escherichia coli *and host urothelial cells when intracellular bacterial communities are assembled J Biol Chem 2007 282 21259 21267 10.1074/jbc.M611502200 17504765

[B19] Mitterhuemer S Petzl W Krebs S Mehne D Klanner A Wolf E Zerbe H Blum H *Escherichia coli *infection induces distinct local and systemic transcriptome responses in the mammary gland BMC Genomics 2010 11 138 10.1186/1471-2164-11-138 20184744 PMC2846913

[B20] Glezer I Chernomoretz A David S Plante MM S R Genes involved in the balance between neuronal survival and death during inflammation PLoS One 2007 2 10.1371/journal.pone.0000310 17375196 PMC1819560

[B21] Lund S Christensen KV Hedtjärn M Mortensen AL Hagberg H Falsig J Hasseldam H Schrattenholz A Pörzgen P Leist M The dynamics of the LPS triggered inflammatory response of murine microglia under different culture and *in vivo *conditions J Neuroimmunol 2006 180 71 87 10.1016/j.jneuroim.2006.07.007 16996144

[B22] Altemeier WA Matute-Bello G Gharib SA Glenny RW Martin TR Liles WC Modulation of lipopolysaccharide-induced gene transcription and promotion of lung injury by mechanical ventilation J Immunol 2005 175 3369 3376 16116230 10.4049/jimmunol.175.5.3369

[B23] Månsson L Melican K Molitoris B Richter-Dahlfors A Progression of bacterial infections studied in real time--novel perspectives provided by multiphoton microscopy Cell Microbiol 2007 2007 10 10.1111/j.1462-5822.2007.01019.x 17662072

[B24] Mobley HLT Green DM Trifillis AL Johnsson DE Chippendale GR Lockatell CV Jones BD Warren JW Pyelonephritogenic *Escherichia coli *and killing of cultured human renal proximal tubular epithelial cells: role of hemolysin in some strains Inf Immun 1990 58 1281 1289 10.1128/iai.58.5.1281-1289.1990 PMC258621 2182540

[B25] Benjamini Y Hochberg Y Controlling the false discovery rate: a practical and powerful approach to multiple testing J R Statist Soc B 1995 57 289 300

[B26] Shannon P Markiel A Ozier O Baliga NS Wang JT Ramage D Amin N Schwikowski B Ideker T Cytoscape: A software environment for integrated models of biomolecular interaction networks Genome Res 2003 13 14597658 10.1101/gr.1239303 PMC403769

[B27] Maere S Heymans K Kuiper M BiNGO: a cytoscape plugin to assess overrepresentation of gene ontology categories in biological networks Bioinformatics applications note 2005 21 3448 3449 10.1093/bioinformatics/bti551 15972284

[B28] Smyth GK Gentleman RC, Carey VJ, Dudoit S, Irizarry R, Huber W Limma: Linear Models for Microarray Data Bioinformatics and computational biology solutions using R and Bioconductor 2005 New York: Springer 397 420 full_text

[B29] Banno T Adachi M Mukkamala L Blumenberg M Unique keratinocyte-specific effects of interferon-g taht protect skin from viruses, identified using transcriptional profiling Antiviral Therapy 2003 8 541 554 14760888

[B30] Sanda C Weitzel P Tsukahar T Schaley J Edenberg HJ Stephens MA Differential gene induction by type I and type II interferons and their combination J Interferon Cytokine Res 2006 26 462 472 10.1089/jir.2006.26.462 16800785

[B31] Reise Sousa C Hieny S Scharton-Kersten T Jankovic D Charest H Germain RN Sher A In vivo microbial stimulation induces rapid CD40 ligand-independent production of interleukin 12 by dendritic cells and their redistribuion to T cell areas J Exp Med 1997 186 1819 1829 10.1084/jem.186.11.1819 9382881 PMC2199158

[B32] van de Wetering D de Paus RA van Dissel JT van de Vosse E IL-23 modulates CD56^+^/CD3^- ^NK cell and CD56^+^/CD3^+ ^NK-like T cell function differentially from IL-12 Int Immunol 2008 21 145 153 10.1093/intimm/dxn132 19088061

[B33] Sutton CE Lalor SJ Sweeney CM Brereton CF Lavelle EC Mills KHG Interleukin-1 and IL-23 induce innate IL-17 production from γδ T cells, amplifying Th17 responses and autoimmunity Immunity 2009 31 331 341 10.1016/j.immuni.2009.08.001 19682929

[B34] Hu X Ivashkiv LB Cross-regulation of signaling pathways by interferon-gamma: implications for immune responses and autoimmune diseases Immunity 2009 31 539 550 10.1016/j.immuni.2009.09.002 19833085 PMC2774226

[B35] Ellis TN Beaman BL Interferon-γ activation of polymorphonuclear neutrophil function Immunol 2004 112 2 12 10.1111/j.1365-2567.2004.01849.x PMC1782470 15096178

[B36] Leto TL Geiszt M Role of nox family of NADPH oxidases in host defense Antioxid Redox Signal 2006 8 1549 1561 10.1089/ars.2006.8.1549 16987010

[B37] Schroder K Hertzog PJ Ravasi T Hume DA Interferon-γ: an overview of, signals, mechanisms and functions J Leukocyte Biol 2004 75 163 189 10.1189/jlb.0603252 14525967

[B38] Li L Huang L Vergis AL Ye H Bajwa A Narayan V Strieter RM Rosin DL Okusa MD IL-17 produced by neutrophils regulates IFN-γ-mediated neutrophil migration in mouse kidney ischemia-reperfusion injury J Clin Invest 2010 120 331 342 10.1172/JCI38702 20038794 PMC2798679

[B39] Sivick KE Schaller MA Smith SN Mobley HL The innate immune response to uropathogenic Escherichia coli involves IL-17A in a murine model of urinary tract infection J Immunol 2010 184 2065 2075 10.4049/jimmunol.0902386 20083670 PMC2821792

[B40] Cray C Zalasa J Altman NH Acute phase response in animals: a review Comp Med 2009 59 517 526 20034426 PMC2798837

[B41] Li X Hassoun HT Santora R Rabb H Organ crosstalk: the role of the kidney Curr Op Crit Care 2009 15 481 487 10.1097/MCC.0b013e328332f69e 19851101

[B42] Miller JH (Ed.) Experiments in molecular genetics 1972 New York: Cold Spring Harbor Laboratory

[B43] Gentleman RC Carey VC Bates DM Bolstad B Dettling M Dudoit S Ellis B Gautier L Ge Y Gentry J Bioconductor: open software development for computational biology and bioinformatics Genome Biology 2004 5 R80 10.1186/gb-2004-5-10-r80 15461798 PMC545600

[B44] kth An R package for analysis of microarray data. R package version 1.2.3 http://www.biotech.kth.se/molbio/microarray/dataanalysis/downl_and_inst.html

[B45] Ashburner M Ball CA Blake JA Botstein D Butler H Cherry JM Davis AP Dolinski K Dwight SS Eppig JT Gene ontology: tool for the unification of biology Nature Genetics 2000 25 25 29 10.1038/75556 10802651 PMC3037419

