# Supplementary material for: Comparative tissue transcriptomics reveal prompt inter-organ communication in response to local bacterial kidney infection
Source: BMC Genomics. 2011 Feb 21;12:123. doi: 10.1186/1471-2164-12-123 (PMC3047304; doi:10.1186/1471-2164-12-123)
Supplement: Additional file 6 — Table of primer sequences used in qRT-PCR [file 1471-2164-12-123-S6.DOC]

| **Primer sequences** | | |
| --- | --- | --- |
| **Rat gene** | **Forward 5’ -> 3’** | **Reverse 5’-> 3’** |
| *Ccl4* | TCCTGCTGCTTCTCTTACAC | TTTGGTCAGAAATACCACAGC |
| *Cd274* | GAAAGTCAACGCTCCATACC | GTCACTGTTTGTCCAGATCAC |
| *Cxcl2* | GTTGACTTCAAGAACATCCAGAG | TTAGCCTTGCCTTTGTTCAG |
| *Gapdh* | GTATGATGACATCAAGAAGGTGG | CATTGTCATACCAGGAAATGAG |
| *Gfp* | ATCCGTTCAACTAGCAGACC | GTTACAAACTCAAGAAGGACCA |
| *Icam1* | TATCGGGATGGTGAAGTCTG | ATTCTGATCATGGTACAGCAC |
| *Ifi47* | TTTGGATCTGATCTTCCTTGGT | GGAGGGCATAATGAATAGCAG |
| *Ifi204* | TGGCTAAGTGTTCAATGGAG | AATGGGTCTGTTGCTTTGAG |
| *Ifit3* | AGTCCTCTGAACTCCTACTC | GCTGTTTAACTTCTTCCTTCTC |
| *Ifng* | GAGCCAGATTATCTCTTTCTACC | GTTGTTCACCTCGAACTTGG |
| *Il6* | GGATATAACCAGGAAATTTGCC | GTATTGCTCTGAATGACTCTGG |
| *Il12a* | GCTAATGCAGTCTCTGAATCAC | GCTCAGATAGTTCATCACCCT |
| *Il12b* | GAGTGTAACCAGAAAGGTGC | TGCATGATGAATTGTAGTAGCG |
| *Il17a* | GTACTCATCCCTCAAAGTTCAG | CACAGAAGGATATCTATCAGGGTC |
| *Il23a* | TGGGACAAATGGATCTACTAAGAG | AGTAGGGAGGTATGAAGCTG |
| *Socs3* | CCCTCCTTCTCTTTACCACC | CCAGAATAGATGTAGTAAGCTCTC |
| *Steap4* | CGAATCGTGTCTTTCCTATAACAG | AGTTAGGGCCTGAGTAATGG |
